# Using Automated Coding of Nonverbal Behavior During a Suicide Assessment to Inform Risk Detection: Mixed Cross-Sectional and Exploratory Prospective Study

**DOI:** 10.2196/85589

**Published:** 2026-09-16

**Authors:** Ilana Gratch, Simon M Li, Yutong Zhu, Alexander Grattery, Jeffrey Cohn, Da-eun (Ellen) Lee, Beatrice Beebe, Christine B Cha

**Affiliations:** 1 Department of Psychiatry, Weill Cornell Medicine, 525 E 68th Street, New York, NY, 10065, United States, (212) 821-0998; 2 Department of Psychological & Brain Sciences, Boston University, Boston, MA, United States; 3 Department of Psychology, Yale University, New Haven, CT, United States; 4 Department of Psychology, The University of Texas at Austin, Austin, TX, United States; 5 Intelligent Systems Program, University of Pittsburgh, Pittsburgh, PA, United States; 6 Department of Psychology, University of Pittsburgh, Pittsburgh, PA, United States; 7 Deliberate AI, New York, NY, United States; 8 Child Study Center, Yale School of Medicine, Yale University, New Haven, CT, United States; 9 New York State Psychiatric Institute, Columbia University Irving Medical Center, New York, NY, United States; 10 Center for Brain and Mind Health, Yale School of Medicine, Yale University, New Haven, CT, United States

**Keywords:** suicide, nonverbal behavior, clinical interview, young adult, automated coding

## Abstract

**Background:**

Suicide assessments have historically privileged verbal report by the patient, despite the fact that nonverbal behaviors of patients and their clinicians may convey important affective and interpersonal information about suicide risk. Recent advances in computational science enable efficient characterization of rich nonverbal data.

**Objective:**

This study aimed to use automated coding to test whether facial action and head motion exhibited by young adults and their clinical interviewers during a widely used suicide assessment can identify suicidal participants.

**Methods:**

Participants were a diverse sample of 66 young adults (age: mean 21.32, SD 2.11 years) recruited from the community, half of whom engaged in past-year suicidal behavior (ie, suicidal participants) and half of whom had no history of suicidality (ie, nonsuicidal participants). Facial action units, head pose, and eye and mouth opening of both participants and clinical interviewers were extracted from the first 3 minutes of a face-to-face, video-recorded Columbia-Suicide Severity Rating Scale (C-SSRS) using the Python-Based Automated Facial Affect Recognition (PyAFAR) software. Nonverbal behaviors of suicidal versus nonsuicidal participants and their interviewers were compared using 2-tailed independent samples *t* tests and Mann-Whitney *U* tests. Binary classification algorithms were then used to test how well these nonverbal behaviors together predicted group membership. Exploratory post hoc analyses assessed whether any nonverbal behaviors at baseline were associated with suicidal participants’ ideation severity or suicidal behavior 3 months later.

**Results:**

Nonverbal behaviors of participants and particularly their clinical interviewers differentiated suicidal versus nonsuicidal young adults at baseline. Suicidal participants demonstrated elevated velocity in opening and closing of eyes and mouth (*P*=.004). Interviewers of suicidal participants showed less animated head movement (*P*=.02), elevated velocity of eye opening and closing (*P*=.02), and ambivalent smiling patterns (*P*s=.01-.046). Overall, interviewer nonverbal behaviors predicted group membership with greater accuracy than participant behaviors, correctly identifying 81% (27/33) versus 59% (19/33) of suicidal young adults. Finally, interviewer smiling occurrence was associated with suicidal participants’ ideation severity (*P*=.04) and suicidal behavior (*P*=.02) 3 months later, while explicit measures, including interviewers’ clinical ratings and participants’ own self-reported ideation severity at baseline, were not.

**Conclusions:**

This study demonstrates the importance of attending to nonverbal channels of communication in suicide assessments, especially those of the clinical interviewer. It also highlights the potential for automated coding to detect clinically meaningful information efficiently and objectively.

## Introduction

### Background

Assessing suicide risk is challenging and consequential. Many patients who die by suicide deny suicidal thinking in their last conversation with a mental health care provider [1,2]. Similarly, many survivors of near-lethal suicide attempts who engaged in treatment at the time of attempt report concealing their intent from their providers beforehand [3]. Even those with more passive ideation tend to disclose and describe their suicidal experiences inconsistently across contexts and assessment methods [4,5]. Yet, to date, clinicians and researchers have had little choice but to rely almost exclusively on what patients say about their suicidal thoughts and behaviors [6].

While these reporting discrepancies may be due in part to fears of the consequences of disclosure (eg, hospitalization [7-9]), they also likely reflect the complexity of the suicidal experience and the limitations of our methods for capturing and quantifying it [6]. Indeed, most suicide assessments rely on responses to yes or no questions, even though many individuals possess ambivalent feelings toward suicide that do not lend themselves to clear-cut, binary responses [6,10]. Further, some experience suicidal thoughts more in the form of imagery than thoughts that can be easily articulated in words [11]. Prior research demonstrates the power of implicit processes pertinent to suicide, such as attentional biases toward suicide and implicit associations with death, both of which have been shown to predict subsequent suicidal thoughts and behaviors [12,13]. Taken together, these findings lend support to the call from Schechter et al [6] for the development of assessments that enable capture of suppressed, dissociated, and/or implicit material related to suicide, as well as calls from researchers for the investigation of markers that capture more than what is voluntarily reported by the patient, such as biochemical and behavioral markers [14].

Nonverbal behavior, the elements of interaction that extend beyond the words spoken, including facial expressions, head movements, gaze patterns, and vocal characteristics, convey important affective and interpersonal information in clinical contexts [15,16]. A growing body of research suggests that nonverbal behaviors, such as the facial action units (AUs) taxonomized in the facial action coding system (FACS) [17,18], contain salient cues in clinical settings. For example, in a study of emergency room patients who recently attempted suicide, patients’ facial expressions during a clinical interview predicted future suicide attempts over the next 2 years [19]. Importantly, these expressions, particularly chin*-*raising, predicted subsequent suicide attempts above and beyond the interviewing psychiatrist’s risk determination [19]. In other studies [20,21], particular AUs and combinations of AUs exhibited across various contexts have differentiated suicidal and nonsuicidal individuals, including inner brow-raisers, outer brow-raisers, brow-lowerers, lip corner-depressors, and Duchenne smiles*,* ie, smiles typically associated with genuine positive affect that involve both the cheek-raiser and the lip corner-puller. One previous study examined not just facial expressions but also head motion exhibited during suicide assessments and found reduced head motion to be associated with more severe suicidal ideation among hospitalized suicidal adults [22]; this same behavior has also emerged as a strong predictor of related psychopathology, such as depression [23]. While the extant research is limited to a small number of studies, and specific findings vary by sample and context, facial action and head motion exhibited during suicide assessments appear to contain rich information.

Decades of research in adjacent disciplines (eg, mother-infant communication) highlight the importance of attending to both members of the interacting dyad when examining nonverbal communication [24]. Yet only one previous study assessed the facial action of the clinical interviewer during an assessment with recent suicide attempters [19]. Results revealed that the interviewing psychiatrist’s facial actions predicted subsequent suicide attempts in the participant, while her written, explicit risk determination did not [19,25]. Similarly, researchers have observed that suicidal adolescents’ clinical interviewers adapt their own vocal prosody in response to suicidal adolescents’ presentations [26]. These findings suggest that important data pertaining to suicide risk may be captured and transmitted via the nonverbal behaviors of both members of the interviewer-participant dyad, warranting further research.

### Machine Learning–Based Facial Action and Head Movement Characterization

Nonverbal behaviors have likely received less attention in clinical research than verbal reports because of the time-intensive and subjective nature of quantifying them [27,28]. Recent advances in machine learning and affective computing afford the opportunity to quantify nonverbal information efficiently and systematically. Deep learning–based approaches, such as the Python-Based Automated Facial Affect Recognition (PyAFAR) software [27,28], enable objective, reliable, and efficient measurement of visual behavior, including facial AUs, head pose, and eye and mouth opening and closing for each frame of video [28]. The continuous, intensive time series data produced makes it possible to characterize dynamic, complex, and out-of-awareness features of the nonverbal channels of communication.

### Study Aims and Hypotheses

#### Overview

No previous studies to our knowledge have harnessed machine learning–based approaches to assess the facial action and head motion of both respondents and interviewers during a face-to-face suicide assessment [19,20,22]. In this study, we use automated coding to characterize facial actions and head motion exhibited during a widely used suicide interview and test whether these nonverbal behaviors can differentiate suicidal and nonsuicidal participants. Specifically, we pursue the following two aims: (1) compare nonverbal behaviors of suicidal and nonsuicidal groups, and (2) predict suicidal versus nonsuicidal group membership.

#### Compare Nonverbal Behaviors of Suicidal and Nonsuicidal Groups

We first test whether nonverbal behaviors of participants and interviewers differ between suicidal and nonsuicidal groups. Specifically, we examine the following nonverbal behaviors of both participants and interviewers: occurrence and intensity of specific facial AUs, velocity of eye and mouth opening and closing, and velocity of head motion. Given the limited existing research, we posed only two a priori hypotheses based on prior findings, including those of a parallel investigation of manually coded AUs in this dataset [21]. We hypothesized: (1) suicidal participants, compared to nonsuicidal participants, will exhibit more eyebrow movement and more oral movement across several AUs (ie, inner brow-raiser, outer brow-raiser, brow-lowerer, lip corner-depressor, and Duchenne smile); and (2) suicidal participants, compared to nonsuicidal participants, will exhibit reduced head motion velocity across the pitch (nod), yaw (turn), and roll (tilt) axes [22]. Although we did not make explicit a priori hypotheses about interviewer behavior due to paucity of research, we explore the same set of nonverbal behaviors among them.

#### Predict Suicidal vs Nonsuicidal Group Membership

We then use machine learning algorithms to predict suicidal versus nonsuicidal group membership from nonverbal behaviors. First, to assess the importance of nonverbal behavior relative to depression severity, we examine differences in the prediction of group membership when integrating nonverbal behavior variables with depression severity, compared to prediction with depression severity alone. Second, to investigate the relative importance of participant versus interviewer nonverbal behaviors, we examine whether participant nonverbal behaviors, or interviewer nonverbal behaviors, better predict suicidal versus nonsuicidal group membership.

## Methods

### Sample and Procedures

Participants were 70 young adults between the ages of 18 and 26 years (mean 21.32, SD 2.11 years) recruited from the community through social media advertisements (eg, Instagram [Meta], Facebook [Meta]) between October 2020 and December 2021. Participants were required to reside in New York State. Participants mostly identified as women. The sample was diverse in terms of race and ethnicity and sexual orientation, with about half the sample identifying as White, and slightly less than half of the sample identifying as heterosexual. See Table 1 for demographic characteristics.

**Table 1.** Participant characteristics (n=66). One participant’s demographic data are missing.

Characteristics	Values
Age (years), mean (SD); min-max	21.32 (2.11); 18-26
Gender identity, n (%)	
Woman	39 (59)
Man	13 (20)
Nonbinary or nonconforming	13 (20)
Sex at birth, n (%)	
Female	53 (80)
Male	12 (18)
Race, n (%)	
White	32 (48)
Black or African American	5 (8)
Asian	20 (30)
Multiracial	6 (9)
Other	2 (3)
Ethnicity (% Hispanic), n (%)	12 (18)
Sexual orientation, n (%)	
Heterosexual or straight	32 (48)
Gay, lesbian, bisexual, or queer	30 (45)
Asexual or other	3 (5)
Lifetime suicidal thoughts and behaviors (% endorsed), n (%)	
Suicidal ideation history	32 (48)
Suicide attempt history	17 (26)
Aborted attempt history	26 (39)
Interrupted attempt history	23 (35)
Preparatory behaviors history	23 (35)
Past-year suicidal behavior (% endorsed), n (%)	
Suicide attempt	5 (8)
Aborted attempt	21 (32)
Interrupted attempt	15 (23)
Preparatory behaviors	16 (24)

Participants were screened for eligibility via an anonymous Qualtrics survey. To be eligible, young adults were required to endorse either: (1) presence of suicidal behavior in the past year (ie, suicide attempt, interrupted attempt, aborted attempt, or preparatory behaviors; n=35); or (2) no lifetime history of suicidal thoughts or behaviors (n=35), both assessed using an online version of the Columbia-Suicide Severity Rating Scale (C-SSRS; [29]). Exclusion criteria were the presence of any factor visibly impairing the young adult’s ability to effectively participate in the study.

Eligible young adults were contacted via email and scheduled for an hour-long online recorded video call. During the video call, master’s-level research assistants administered the C-SSRS interview as well as a brief self-report battery via Qualtrics (see Measures). Any participant reporting a history of suicidal or self-injurious thoughts or behaviors at any point during the call received a risk assessment and safety plan prior to the termination of the video call.

Suicidal participants were contacted via email 3 months following the baseline interview and were invited to complete an online self-report survey administered via Qualtrics. The follow-up survey assessed the presence or absence of suicidal thoughts and behaviors over the past 3 months as well as past-month suicidal ideation severity. Of the 33 suicidal participants in our analytic sample, 28 completed the follow-up survey and were included in our post hoc exploratory prospective analyses.

### Ethical Considerations

All participants provided informed consent at the start of the video call. Deception was used regarding the rationale for the video recording to minimize potential bias in nonverbal behaviors. Participants were initially informed that the video recordings were for response verification and training purposes. Full disclosure regarding the study’s true purpose was provided at the conclusion of the study, at which point participants had the option to withdraw and request video deletion. Video recordings were stored on a secure, Health Insurance Portability and Accountability Act (HIPAA)–compliant drive using a unique ID number, distinct from ID numbers used for all other participant data. All participants were compensated with a US $25 Amazon gift card. Participants who completed the 3-month follow-up online survey were compensated with an additional US $10 Amazon gift card. All study procedures were approved by the Teachers College, Columbia University Institutional Review Board (IRB #s 20‐274, 22‐135, 23‐260).

### Measures

#### C-SSRS

The C-SSRS [29] is a widely used interview that assesses lifetime and recent self-injurious thoughts and behaviors, including passive death wishes, active suicidal ideation, intent, method, plan, preparatory behaviors, aborted attempts, interrupted attempts, actual attempts, and nonsuicidal self-injury. The C-SSRS was used twice in this study. First, in the eligibility screener, participants were administered an online, self-report survey version of the C-SSRS to determine the presence versus absence of past-year suicidal behavior. Second, the C-SSRS was administered in its full form by trained, master’s-level clinical interviewers during the face-to-face interview. Interviewers asked about past month and lifetime suicidal thoughts, and past 3 months and lifetime suicidal behavior. Participant and interviewer nonverbal behaviors were extracted from the first 3 minutes of the video-recorded C-SSRS interview at baseline. Research suggests that “thin slices” of social behavior, as brief as 30 seconds, are no less informative than longer observations [30].

#### Nonverbal Behaviors

Nonverbal behaviors exhibited by both participants and clinical interviewers during the first 3 minutes of the C-SSRS were extracted using the PyAFAR software [27,31]. PyAFAR is a Python-based, open-source, out-of-the-box software for automatic AU detection, with a built-in pipeline that includes four components: (1) face tracking and head orientation; (2) face registration; (3) AU detection; and (4) visualization. The software is described in detail by its creators elsewhere [27]. PyAFAR was used to determine frame-by-frame occurrence and intensity of facial AUs, eye aspect ratio (EAR), mouth aspect ratio (MAR), and head positioning for each frame of video. We use tsfresh [32], a Python package for systematic feature engineering for time-series data, to determine more dynamic summary statistics that characterize our data at the group level, described below.

#### Facial Expressions

**Figure 1.**
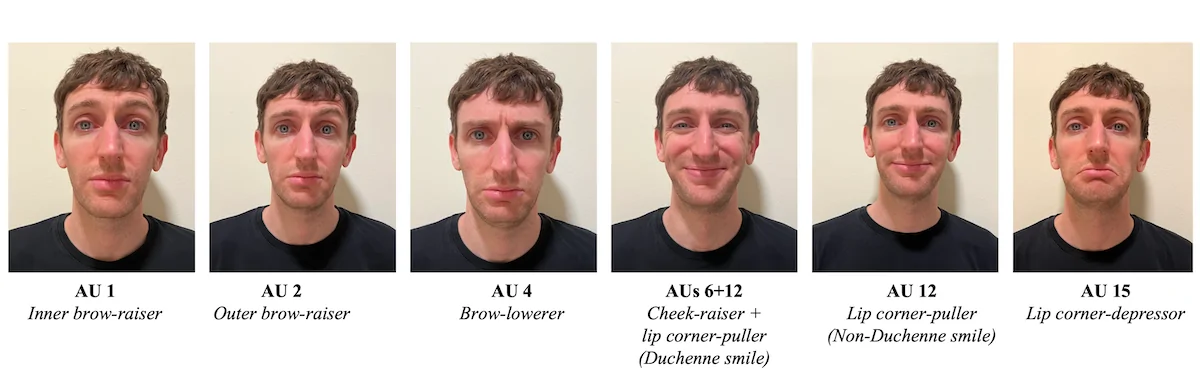
Demonstration of action units in the facial action coding system. AU: action unit.

Using PyAFAR, we extract the probability of occurrence of each of the following AUs for each frame of video: 1 (inner brow-raiser), 2 (outer brow-raiser), 4 (brow-lowerer), 6 (cheek-raiser), 12 (lip corner-puller), 15 (lip corner-depressor), and 17 (chin-raiser). See Figure 1. A threshold of 0.5 was used to determine AU occurrence in a given frame [27]. We determine AU occurrence by calculating the proportion of frames in which each AU occurred. We also extract intensity ratings on a 0‐5 scale for AUs 6 and 12 (1=trace-level; 5=most intense). Consistent with previous research [33], after recoding 1 s as 0 s, we calculate time-series features including mean (SD) and complexity (ie, a measure of the number of peaks and valleys in intensity throughout the 3 min) using tsfresh [32]. Aggregates of each of these features are presented at the group level.

We also extract frame-by-frame EAR, a measure capturing the ratio of the distance between upper and lower eyelid landmarks to the distance between eye corners, as well as MAR, the equivalent for the upper and lower lips and mouth corners [32]. We calculate frame-by-frame EAR and MAR displacement and velocity and use the root mean square of the latter as an overall measure of velocity of eye and mouth opening and closing. Additional time-series features related to eye opening and closing were extracted from tsfresh based on findings in prior research, including eye opening and closing permutation entropy and eye opening and closing change in quantile of mean [32,34], also presented at the group level in the Results. Permutation entropy captures predictability versus randomness of patterns in time series data. Change in quantile of mean is calculated across multiple fixed corridors which are determined based on the lower and higher quantiles of the distribution of the time series data. The average absolute value of consecutive changes of the time series inside each corridor is then calculated [33]. Because there are 15 summary statistics within our data comparing various corridors on the change in quantile of mean, we correct for multiple comparisons in these analyses using a Bonferroni correction.

#### Head Motion

We use head positioning in each frame of video, provided by PyAFAR, across 3 axes: pitch (nod), yaw (turn), and roll (tilt) to determine head motion velocity. We first calculate each participant’s mean head positioning, then determine frame-by-frame angular displacement, and finally calculate the derivative of displacement across each axis [35]. The root mean square of each axis was then used as an overall summary statistic of head motion velocity for pitch, yaw, and roll.

#### Suicidal Ideation Questionnaire

Suicidal ideation severity at baseline and the 3-month follow-up was assessed with the Suicidal Ideation Questionnaire (SIQ; [36]), a self-report measure containing 30 items rated on a 6-point scale with high internal consistency and average test-retest reliability and construct validity, including in our sample (*α*=.98). The SIQ is scored by summing responses to each of the items and has a clinical cut-off value of 41 [36].

#### Quick Inventory of Depressive Symptomatology

The 16-item self-report version of the Quick Inventory of Depressive Symptomatology (QIDS [37]) was used to assess current depression severity (*α*=.88) at baseline. Items are rated on a scale of 0 to 3, and overall scores are determined by summing each value [37]. To better isolate depression severity, the item assessing suicidal ideation severity was removed from the overall QIDS scores used in binary classification algorithms.

### Statistical Analyses

#### Intersystems Reliability

Approximately 20% of videos were coded by certified human FACS coders to assess the performance of PyAFAR on our data with regard to AU detection. The reliability dataset consisted of 37,645 frames across 26 unique videos (30 fps) that were coded by both PyAFAR and a human FACS coder. Because human coders record the occurrence of AUs at the millisecond level, and PyAFAR produces a probability of occurrence for each frame of video, we transformed the output of both to presence or absence of each AU for each tenth of a second. For each AU, we determined the overall, chance-adjusted S-Score, the area under the curve (AUC), positive agreement (ie, *F*_1_-score), and negative agreement using R (Table 2) [38]. AUs with an overall, chance-adjusted S-score of .70 or greater are included in subsequent analyses, including AUs 1, 4, 6, 12, and 15. Overall, negative agreement was much stronger than positive agreement across AUs, likely attributable to low AU occurrence rates in the reliability dataset.

**Table 2.** Python-Based Automated Facial Affect Recognition (PyAFAR) versus human coder intersystem reliability. This table displays several metrics assessing intersystem reliability between PyAFAR’s automated coding and human coding completed by certified FACS^a^ coders.

Action unit	S-score (95% CI)	AUC^c^	Positive agreement or *F*_1_-score	Negative agreement
AU 1 (inner brow-raiser)^b^	0.71 (0.70-0.72)	0.60	0.28	0.92
AU 2 (outer brow-raiser)	0.46 (0.45-0.47)	0.64	0.27	0.83
AU 4 (brow-lowerer)^b^	0.95 (0.95-0.95)	0.50	0.02	0.99
AU 6 (cheek-raiser)^b^	0.83 (0.82-0.83)	0.74	0.12	0.95
AU 12 (lip corner-puller)^b^	0.76 (0.76-0.77)	0.58	0.22	0.94
AU 15 (lip-depressor)^b^	0.93 (0.93-0.94)	0.61	0	0.98
AU 17 (chin-raiser)	0.68 (0.68-0.69)	0.61	0	0.91

a FACS: facial action coding system.

b Action unit (AU) included in subsequent analyses.

c AUC: area under the curve.

#### Aims

The statistical analysis for our primary aims were as follows:

1. Compare nonverbal behaviors of suicidal and nonsuicidal groups: to compare suicidal versus nonsuicidal participants on key demographic characteristics including age, race, sex at birth, and gender identity, we conducted independent samples 2-tailed *t* tests (effect size: Cohen *d* and its 95% CI) and chi-square tests (effect size: Cramér V and Φ). To compare suicidal versus nonsuicidal participant nonverbal behaviors, we conducted independent samples 2-tailed *t* tests (effect size: Cohen *d* and its 95% CI) and Mann-Whitney *U* tests when Levene tests indicated the presence of unequal variance between groups (effect size: *r* and 95% bootstrapped CI with 5000 resamples using NumPy [39] and SciPy [40]). We repeat the same group comparison analyses to test whether interviewer nonverbal behaviors differed in interviews with suicidal versus nonsuicidal participants.
2. Predict suicidal versus nonsuicidal group membership: we then used binary classification algorithms to test how well these nonverbal behaviors together predict group membership. We evaluated both logistic regression (LogisticRegression) and support vector machine (SVM; LinearSVC) using scikit-learn in Python [41]. Overall, across models run, SVM performed slightly better, likely because it is well suited to smaller datasets. Therefore, we present only the results of SVM models.

For model optimization, hyperparameters were tuned using a grid search based on leave-one-out cross-validation (LOOCV). The optimal hyperparameters identified were: a maximum of 2000 iterations, the “linear” kernel, 0.5 for the tolerance for stopping criteria, and default values in *s*cikit-learn. Before training, all features were normalized using the Robust Scaler, which is based on percentiles and therefore unaffected by a small number of extreme outliers [41]. We evaluate the ability of the following models to predict suicidal versus nonsuicidal group membership: (1) depression severity: a model containing participant depression severity only; (2) depression severity + significant participant and interviewer nonverbals: a model containing depression severity plus participant and interviewer nonverbal behaviors deemed to be significant in group comparisons; (3) all participant nonverbals: a model containing all participant nonverbal behaviors; (4) all interviewer nonverbals: a model containing all interviewer nonverbal behaviors; (5) significant participant nonverbals: a model containing participant nonverbal behaviors deemed significant in group comparisons; and (6) significant interviewer nonverbals: a model containing interviewer nonverbal behaviors deemed significant in group comparisons. See Textbox 1 for a list of features included in each model. We omit change in quantile of mean and permutation entropy variables in these models to reduce the number of predictors in the model due to small sample size.

Textbox 1. **Features included in support vector machine models.**

**All participant**

- Pitch velocity
- Yaw velocity
- Roll velocity
- Action unit (AU) 1 occurrence
- AU 4 occurrence
- AU 12 occurrence
- AUs 6+12 occurrence
- AU 15 occurrence
- Eye aspect ratio velocity
- Mouth aspect ratio velocity
- AU 6 intensity – mean
- AU 6 intensity – SD
- AU 6 intensity – complexity

**All interviewer**

- Pitch velocity
- Yaw velocity
- Roll velocity
- AU 1 occurrence
- AU 4 occurrence
- AU 12 occurrence
- AUs 6+12 occurrence
- AU 15 occurrence
- Eye aspect ratio velocity
- Mouth aspect ratio velocity
- AU 6 intensity – mean
- AU 6 intensity – SD
- AU 6 intensity – complexity

**Significant participant**

- Eye aspect ratio velocity
- Mouth aspect ratio velocity

**Significant interviewer**

- Pitch velocity
- Yaw velocity
- AU 12 occurrence
- AUs 6+12 occurrence
- Eye aspect ratio velocity
- AU 6 intensity - mean
- AU 12 intensity - complexity

We evaluate the performance of each model using LOOCV and compute the average scores for the following metrics: accuracy (proportion correctly classified overall), precision (proportion of suicidal cases correctly classified out of all cases classified as suicidal), sensitivity (proportion of suicidal cases correctly classified out of all actual suicidal cases), F1-score (balance between precision and recall), specificity (proportion of nonsuicidal cases correctly classified as nonsuicidal), and receiver operating characteristic area under the curve (ROC AUC; balance between sensitivity and specificity where 1.0=perfect discrimination, 0.5=no better than chance).

Data were analyzed in IBM SPSS Statistics, R, and Python. Study hypotheses, informed by results of manual FACS coding of an overlapping but distinct excerpt of the C-SSRS examined here, and analysis plan were preregistered following data collection on the Open Science Framework [21,42]. See Multimedia Appendix 1 for a detailed overview of deviations from our preregistration. With regard to missing data, 3 participant and interviewer video recordings and 1 interviewer recording were not accessible due to a file-saving error, and one participant withdrew partly through the study; video data were not retained for this participant or interviewer. These participants were excluded from analyses. One participant’s demographic data are missing.

### Post Hoc Exploratory Analyses: Prospective Suicidal Thoughts and Behaviors

We pursued post hoc exploratory analyses testing whether any participant and interviewer nonverbal behaviors that differentiated groups at baseline were associated with ideation severity and/or suicidal behavior 3 months later among a subset of suicidal participants who completed a follow-up survey 3 months later. We use Mann-Whitney *U* tests, 2-tailed independent samples *t* tests, and bivariate correlation, as appropriate, and do not run predictive models due to the small sample size for these exploratory analyses (n=28). For comparison, we also test whether participants’ baseline ideation severity on the SIQ, or interviewers’ clinical ratings, predict the same. Clinical ratings were comprised of quantitative risk level assignments that interviewers made immediately following the interview based on information gathered and clinical intuition, ranging from 1 (“No risk at all”) to 5 (“Very high risk”). We do not run predictive models due to the small sample size for these exploratory analyses (n=28).

## Results

### Sample Characteristics

The final analytic sample for whom we retained video recordings comprised 66 participants (Table 1). The analytic sample included 33 suicidal participants and 33 nonsuicidal participants. Lifetime suicidal behaviors reported by suicidal participants included: suicide attempts (n=17, 26% of participants), interrupted attempts (n=23, 35%), aborted attempts (n=26, 39%), and preparatory behaviors (n=23, 35%). With regard to severity of current suicidal ideation and depressive symptoms, the mean ideation severity among suicidal participants was 69.97 (SD 34.71), compared to 9.61 (SD 7.55) among nonsuicidal participants and a clinical cut-off value of 41 [36]. Mean depression severity among suicidal participants fell in the “moderate” range (mean 12.65, SD 5.39), and in the “none/mild” range among nonsuicidal participants (mean 5.61, SD 3.90) [37]. Suicidal and nonsuicidal groups did not differ on age (*t*_62_=1, Cohen *d*=0.25, 95% CI −0.24 to 0.74; *P*=.32), sex at birth (*χ*^2^_1_=0.41, *Φ*=.08; *P*=.52), or racial composition (*χ*^2^_4_=6.45, Cramér V=.32; *P*=.17). Gender identity differed significantly between groups (*χ*^2^_2_=13.36, Cramér V=.46; *P*=.001), with the suicidal group comprising more men (n=8) and gender nonconforming and gender queer individuals (n=11) and fewer women (n=13), relative to the nonsuicidal group (men=5; gender nonconforming=1; women=26).

### Differentiating Suicidal and Nonsuicidal Young Adults

#### Group Comparisons: Participant Nonverbal Behaviors

As demonstrated in Table 3, and in contrast with hypotheses, there were no significant differences between suicidal and nonsuicidal participants in occurrence or intensity of any of the AUs measured during the interview. Suicidal participants exhibited significantly greater EAR and MAR velocity (Table 3). EAR velocity permutation entropy did not significantly differ between suicidal and nonsuicidal participants (*t*s=−0.03 to −0.42; *P*s=.780-.976). EAR velocity change in quantile of the mean differed significantly between suicidal and nonsuicidal participants across 14 of the 15 corridors tested. After correcting for multiple comparisons (*α*=.0033), 11 of the corridor comparisons remained significant, with suicidal participants (mean: range 0.007‐0.017) exhibiting significantly greater changes in mean quantile across corridors compared to nonsuicidal participants (mean: range 0.005‐0.015; *t*s_64_=−3.74 to –1.81, *P*s=<.001-.075). Contrary to hypotheses, there were no statistically significant differences in participant head motion velocity across pitch, yaw, and roll (Table 3).

**Table 3.** Participant facial expression and head motion during a suicide assessment (n=66).

Facial expression and head motion	Suicidal participants	Nonsuicidal participants	Test statistic^a^	Effect size (95% CI)
AU^b^ 1^c^, frames present (%)	5.72	1.45	*U*=443.50 (*P*=.17)	*r*=0.16 (−0.07 to 0.38)
AU 4^d^, frames present (%)	0.31	0.78	*U*=535.50 (*P*=.91)	*r*=−0.01 (−0.24 to 0.23)
AUs 6+12^e^, frames present (%)	2.36	2.07	*U*=524 (*P*=.79)	*r*=0.03 (−0.21 to 0.28)
AU 12^f^, frames present (%)	9.10	6.83	*U*=450 (*P*=.23)	*r*=0.15 (−0.09 to 0.38)
AU 15^g^, frames present (%)	0.74	4.67	*U*=537 (*P*=.92)	*r*=0.01 (−0.22 to 0.25)
AU 6^h^, mean intensity (SD)	0.06 (0.14)	0.07 (0.13)	*t*=0.34 (*P*=.73)	*d*=0.08 (−0.40 to 0.57)
AU 12, mean intensity (SD)	0.20 (0.36)	0.17 (0.23)	*t*=−0.53 (*P*=.60)	*d*=−0.13 (−0.61 to 0.35)
AU 6, mean SD of intensity (SD)	0.26 (0.24)	0.28 (0.27)	*t*=0.27 (*P*=.79)	*d*=0.07 (−0.42 to 0.55)
AU 12, mean SD of intensity (SD)	0.40 (0.34)	0.42 (0.29)	*t*=0.19 (*P*=.85)	*d*=0.05 (−0.44 to 0.53)
AU 6, mean complexity of intensity (SD)	70.71 (19.83)	61.02 (27.99)	*t*=−1.62 (*P*=.11)	*d*=−0.40 (−0.89 to 0.09)
AU 12, mean complexity of intensity (SD)	59.82 (20.72)	60.45 (21.18)	*t*=0.12 (*P*=.90)	*d*=0.03 (−0.45 to 0.51)
Velocity				
EAR^i^	0.98	0.77	*U*=318 (*P*=.004)	*r*=0.36 (0.13 to 0.56)
MAR^j^	1.18	0.43	*U*=154 (*P*<.001)	*r*=0.62 (0.45 to 0.75)
Head pitch, RMS^k^ median (IQR)	3.29 (1.69)	3.96 (5.45)	*U*=466 (*P*=.31)	*r*=−0.12 (−0.36 to 0.12)
Head yaw, RMS median (IQR)	5.48 (4.24)	7.52 (7.85)	*U*=438 (*P*=.17)	*r*=−0.17 (−0.40 to 0.07)
Head roll, RMS median (IQR)	0.57 (0.28)	0.60 (0.39)	*U*=512 (*P*=.68)	*r*=0.05 (−0.19 to 0.29)

a For all *t* tests, *df*=64.

b AU: action unit.

c AU 1=inner brow-raiser.

d AU 4=brow-lowerer.

e AUs 6+12=Duchenne smile.

f AU 12=lip corner-puller.

g AU 15=lip corner-depressor.

h AU 6=cheek-raiser.

i EAR: eye aspect ratio.

j MAR: mouth aspect ratio.

k RMS: root mean square.

#### Group Comparisons: Interviewer Nonverbal Behaviors

There were no significant differences in interviewer demonstrations of AUs 1, 4, 12, or 15 with suicidal versus nonsuicidal participants (Table 4). However, interviewers exhibited significantly fewer Duchenne smiles (ie, AUs 6+12 together) and lip corner-pullers (ie, AU 12) with suicidal participants compared to nonsuicidal participants, as well as greater complexity in the intensity of their lip corner-pullers (ie, AU 12) with suicidal versus nonsuicidal participants (Table 4). Interviewers also displayed significantly reduced intensity, on average, as well as less variable (ie, lower SD) frame-by-frame intensity, in their cheek-raisers (AU 6) with suicidal versus nonsuicidal participants (Table 4). Interviewers exhibited significantly greater EAR velocity with suicidal participants versus nonsuicidal participants (Table 4). There were no differences in interviewer MAR velocity, EAR velocity permutation entropy, or EAR velocity change in quantile of mean across any of the corridors after controlling for multiple comparisons (Table 4). With regard to head motion, interviewers exhibited significantly reduced pitch and yaw velocity with suicidal versus nonsuicidal participants, while head roll velocity did not differ between groups (Table 4).

**Table 4.** Interviewer facial expression and head motion during a suicide assessment (n=65).

Facial expression and head motion	Interviewers with suicidal participants	Interviewers with nonsuicidal participants	Test statistic^a^	Effect size (95% CI)
AU^b^ 1^c^, frames present (%)	8.66	17.63	*U*=508.50 (*P*=.79)	*r*=–0.03 (–0.28 to 0.21)
AU 4^d^, frames present (%)	0.29	0.48	*U*=408.00 (*P*=.11)	*r*=–0.20 (–0.43 to 0.03)
AUs 6+12^e^, frames present (%)	0.97	2.73	*U*=350.50 (*P*=.02)^f^	*r*=–0.29 (–0.51 to –0.05)
AU 12^g^, frames present (%)	2.47	5.08	*U*=365.50 (*P*=.03)^f^	*r*=–0.26 (–0.49 to –0.02)
AU 15^h^, frames present (%)	4.35	1.89	*U*=518.50 (*P*=.89)	*r*=–0.02 (–0.24 to 0.22)
AU 6^i^, median intensity (IQR)	0.01 (0.03)	0.03 (0.13)	*U*=336.50 (*P*=.01)^f^	*r*=–0.31 (–0.53 to –0.07)
AU 12, median intensity (IQR)	0.26 (0.38)	0.17 (0.60)	*U*=495.50 (*P*=.67)	*r*=–0.05 (–0.29 to 0.21)
AU 6, median SD of intensity (IQR)	0.14 (0.20)	0.29 (0.49)	*U*=334.50 (*P*=.01)^f^	*r*=–0.32 (–0.53 to –0.08)
AU 12, median SD of intensity (IQR)	0.68 (0.59)	0.57 (0.71)	*U*=486.50 (*P*=.59)	*r*=–0.07 (–0.30 to 0.19)
AU 6, mean complexity of intensity (SD)	69.87 (27.85)	57.51 (27.16)	*t*=–1.81 (*P*=.08)	*d*=–0.45 (–0.94 to 0.05)
AU 12, mean complexity of intensity (SD)	63.07 (19.04)	53.12 (20.28)	*t*=–2.04 (*P*=.046)^f^	*d*=–0.51 (–0.99 to –.01)
Velocity				
EAR^k^, mean RMS^j^ (SD)	0.71 (0.15)	0.62 (0.14)	*t*=–2.42 (*P*=.02)^f^	*d*=−0.60 (−1.10 to –0.10)
MAR^l^, mean RMS (SD)	1.14 (0.40)	1.27 (0.45)	*t*=1.20 (*P*=.24)	*d*=0.30 (−0.19 to 0.78)
Velocity				
Head pitch, median RMS (IQR)	3.09 (0.79)	3.66 (5.86)	*U*=343 (*P*=.02)^f^	*r*=−0.30 (−0.52 to –0.07)
Head yaw, median RMS (IQR)	4.63 (2.44)	5.57 (10.31)	*U*=352 (*P*=.02)^f^	*r*=−0.29 (−0.50 to –0.05)
Head roll, median RMS (IQR)	0.58 (0.41)	0.71 (0.62)	*U*=397 (*P*=.09)	*r*=−0.21 (−0.44 to 0.03)

a For all *t* tests, *df*=63.

b AU: action unit.

c AU 1=Inner brow-raiser.

d AU 4=Brow-lowerer.

e AU 6+12=Duchenne smile.

f Significant at *P*<.05.

g AU 12=lip corner-puller.

h AU 15=lip corner-depressor.

i AU 6=cheek-raiser.

j RMS: root mean square.

k EAR: eye aspect ratio.

l MAR: mouth aspect ratio.

#### Predicting Group Membership: Using Depression Severity and Adding Nonverbal Behavior

Our SVM classifier using depression severity alone (ie, depression severity in Table 5) performed well in its ability to distinguish groups, correctly classifying 80% (52/65) of participants overall (AUC=0.78). However, the model containing selected participant and interviewer nonverbal behaviors in addition to depression severity (ie, Dep *+* Sig Par and Int Nvbs in Table 5) performed better across accuracy, precision, sensitivity, *F*_1_-score, and ROC AUC, correctly classifying 86% (56/65) of participants overall (AUC=0.93). The latter model, including nonverbal behaviors, was especially strong in its ability to identify suicidal individuals in particular, with a sensitivity of 0.88 (29/33) versus 0.75 (25/33) using depression severity alone.

**Table 5.** Support vector machine models predicting suicidal versus nonsuicidal group membership (n=65). The six models included predict suicidal versus nonsuicidal group membership using: (1) depression severity alone; (2) depression severity and significant participant and interviewer nonverbal behaviors; (3) all participant nonverbal behaviors; (4) all interviewer nonverbal behaviors; (5) all significant participant nonverbal behaviors; and (6) all significant interviewer nonverbal behaviors.

Models	Accuracy	Precision	Sensitivity	*F*_1_-score	Specificity	ROC AUC^a^
Depression severity (Dep)	0.80	0.83	0.75	0.79	0.85	0.78
Dep + Sig Par and Int Nvbs	0.86	0.85	0.88	0.86	0.85	0.93
All Nvbs - Par	0.68	0.70	0.59	0.66	0.76	0.79
All Nvbs - Int	0.77	0.74	0.81	0.78	0.73	0.86
Sig Nvbs - Par	0.71	0.76	0.59	0.67	0.82	0.84
Sig Nvbs - Int	0.71	0.67	0.81	0.73	0.61	0.84

a ROC AUC: receiver operating characteristic area under the curve.

#### Predicting Group Membership: Participant vs Interviewer Nonverbal Behaviors

Our SVM classifier containing all interviewer nonverbal behaviors (ie, All Nvbs - Int in Table 5) performed better than the classifier containing all participant nonverbal behaviors (ie, All Nvbs - Par in Table 5) across all metrics, with the interviewers’ nonverbal behaviors correctly classifying 77% (50/65) of participants (vs 68% [44/65] using participants’ own nonverbal behaviors) and correctly identifying 81% (27/33) of suicidal participants in particular (vs 59% [19/33] using their own nonverbal behaviors). SVMs containing significant participant nonverbal behaviors (ie, Sig Nvbs – Par in Table 5) and significant interviewer nonverbal behaviors (ie, Sig Nvbs – Int in Table 5) performed similarly in their ability to predict group membership, with both models correctly classifying 71% (46/65) of participants. The model containing significant interviewer nonverbal behaviors performed better in the identification of suicidal participants in particular (81% [27/33] vs 59% [19/33] using participant nonverbal behaviors).

### Post Hoc Exploratory Analyses: Prospective Suicidal Thoughts and Behaviors

Mean suicidal ideation severity at follow-up (n=28) was 55.46 (SD 22.15), compared to 63.85 (SD 29.96) among the same group of participants at baseline (*t*_26_=1.55; *P*=.13). Of these, 29% (8/28) reported engaging in suicidal behavior since baseline. Suicidal behaviors reported included preparatory behaviors (n=4), aborted attempt (n=3), and interrupted attempt (n=1). Due to small sample sizes, a composite variable was created and recoded as 3-month suicidal behavior (presence vs absence).

Participant nonverbal behaviors that differentiated suicidal and nonsuicidal participants at baseline (ie, EAR and MAR velocity) were not associated with subsequent suicidal ideation severity (*r*_EAR_=−0.23, *P*=.19, *r*_MAR_=−0.03; *P*=.89) or suicidal behavior (*t*_EAR_(25)=−1.27, *P*=.22, *U*_MAR_=41, *z*=−1.61; *P*=.12).

However, interviewer nonverbal behavior during the baseline C-SSRS was associated with subsequent suicidal ideation severity and suicidal behavior. We examined the 3 domains of interviewer behavior that differentiated suicidal and nonsuicidal participants at baseline (ie, head motion velocity, EAR velocity, and smiling) and found that interviewer lip corner pullers (ie, AU 12) during the C-SSRS were associated with participants’ suicidal ideation severity and behavior 3 months postbaseline, such that interviewers demonstrated more lip corner-pullers with participants who reported more severe suicidal ideation at follow-up (*r*=.38; *P*=.04) and who engaged in subsequent suicidal behavior (*U=33, z*=−2.39; *P*=.02). Of note, explicit clinical risk assessment ratings made by the interviewers immediately following the interview were not associated with participants’ subsequent suicidal behavior (*t*_26_=−1.27; *P*=.22) or subsequent suicidal ideation severity (*r*=−0.04; *P*=.86). Participants’ baseline ideation severity was also not associated with subsequent suicidal behavior (*t*_25_=−1.74; *P*=.09) or subsequent suicidal ideation severity (*r*=0.33; *P*=.09).

## Discussion

### Principal Findings

We used machine learning–based tools to characterize nonverbal behaviors of both participants and their clinical interviewers during a face-to-face C-SSRS video interview with suicidal and nonsuicidal young adults. Our primary aims were to test (1) whether nonverbal behaviors of participants and interviewers differ in interviews with suicidal versus nonsuicidal young adults and (2) the extent to which nonverbal behaviors can be used to predict group membership. Finally, in a post hoc exploratory analysis, we tested whether any participant or interviewer nonverbal behaviors that differentiated groups at baseline were associated with participants’ subsequent suicidal thoughts and behaviors 3 months later.

This study represents the first effort to our knowledge to characterize both participant and interviewer facial action and head motion using automated coding during a suicide assessment [19,20,22]. While nonverbal behaviors of both young adults and their interviewers differentiated suicidal (vs nonsuicidal) participants, we documented that interviewers’ nonverbal behaviors reflected differences across more dimensions of nonverbal behavior, were just as (if not more) discriminating, and corresponded to participants’ suicidal ideation and behavior 3 months later. In our study, the distress of the participant is thus more readily detected through the clinical interviewer.

### Nonverbal Behaviors Differ in Interviews With Suicidal vs Nonsuicidal Participants

#### Participant Nonverbal Behaviors

Among participants, only eye and mouth opening and closing velocity differed between suicidal versus nonsuicidal groups. In contrast to our hypotheses and the limited prior research, suicidal participants did not differ from nonsuicidal participants on any specific facial AUs measured [43] or on their head motion velocity [22]. However, suicidal participants did exhibit greater velocity in both eye and mouth opening and closing, and greater change in velocity in eye opening and closing. Elevated velocity in eye opening and closing may be reflective of greater emotional arousal among suicidal participants during the interview. Previous research suggests velocity of eye opening and closing may be linked to underlying fear states [44] and has been linked to depression severity [32].

#### Interviewer Nonverbal Behaviors

##### Overview

Interviewer nonverbal behaviors during interviews with suicidal versus nonsuicidal young adults differed across more domains than participants’ own nonverbal behaviors, namely head movement, eye opening and closing, and smiling.

##### Head Movement

With suicidal participants, interviewer head movement was less animated across both the horizontal (pitch) and vertical (yaw) axes. It is only in the past decade that affective computing research has taken an interest in head motion (beyond controlling for it in facial expression analyses), and this growing body of work documents that head movement contains salient intra- and interpersonal information [35,45,46]. Samanta and Guha [47] argue that head motion alone contains rich information about emotion: it is faster during anger and joy, and reduced during sadness, surprise, and neutral states. It is likely that reduced head motion velocity in our interviewers captured some distressed aspect of their emotional state, such as sadness, surprise, or fear, reflecting a stabilization and freezing that occurred while they learned about participants’ suicide histories as the interview unfolded.

##### Eye Opening and Closing

Similar to the participants, interviewers also showed a higher rate of change (velocity) in their eye opening and closing with suicidal participants, potentially associated with emotional arousal and fear [44]. This finding is similar to previous research which found frequency and intensity of the interviewer’s periocular activation predicted the patient’s subsequent suicide attempt, a pattern they attribute to greater anxiety and preoccupation [19,25].

##### Smiling

With suicidal participants, interviewers engaged in less frequent lip corner pulling and less frequent Duchenne smiling (ie, smiles which include zygomatic major muscle movement, which wrinkles the eyes, and are typically found in moments of genuine positive affect). At the same time, their mouth widening movements showed more modulation (more frequent widening and narrowing of their smiles) with suicidal participants compared to nonsuicidal participants, perhaps indicative of a regulatory process in response to their own distress.

Taken together, interviewers’ less animated head movement, suggesting vigilance; elevated velocity of eye opening and closing, suggesting emotional arousal; and ambivalent mouth widening and narrowing patterns contribute to a picture of the interviewer of the suicidal participant in a complex, distressed state. Moreover, this distressed state is communicated through multiple nonverbal modalities: head, eye, and smile.

### Nonverbal Behaviors of Participants and Especially Interviewers Predict Group Membership

While depression severity alone was a strong predictor of suicidal versus nonsuicidal group membership (correctly classifying 80% [52/65] of participants), the inclusion of important nonverbal behaviors of participants and interviewers improved classification accuracy slightly (86% [56/65]). Importantly, the integration of these nonverbal behaviors was especially critical for the detection of suicidal young adults; while depression severity alone correctly identified 75% (25/33) of suicidal participants, the model incorporating participant and interviewer nonverbal behaviors captured 88% (29/33). Our findings also suggest that interviewer nonverbal behaviors are particularly informative in predicting suicidal versus nonsuicidal group membership of participants; across all participant and interviewer nonverbal behaviors assessed, the model containing all interviewer nonverbal behaviors better predicted group membership than did the model containing all participant nonverbal behaviors: interviewer nonverbal behaviors alone identified 81% (27/33) of suicidal participants, while participant nonverbal behaviors alone captured 59% (19/33).

The importance of interviewer nonverbal behavior is further underscored by our exploratory prospective analyses, which revealed that interviewer nonverbal behavior, but not participant nonverbal behavior, was associated with participant suicidal ideation and behavior 3 months later. Strikingly, neither interviewer clinical ratings of participant suicide risk nor participant self-report of ideation severity were associated with suicidal thoughts or behavior 3 months later. Thus, neither interviewer nor participant seemed consciously aware of suicide risk. These interviewer findings replicate for the first time the seminal work of Heller et al [19], who found that an emergency room psychiatrist’s implicit, out-of-awareness nonverbal behavior predicted which of her patients went on to reattempt suicide over the next two years, while her explicit, written risk predictions did not. In both studies, the interviewer knew with her body what she did not know with her words.

In our study, interviewers’ more frequent use of the lip corner-puller (but not the Duchenne smile) was associated with participant suicidal ideation severity and behavior 3 months later. Non-Duchenne smiles, which contain only the lip corner-puller, have historically been referred to as the “masking smile,” “false smile,” “anticipatory smile,” and “miserable smile,” as they are widely thought to conceal negative affect [48]. Although the current state of research on the distinction between Duchenne and non-Duchenne smiling is mixed [49], non-Duchenne smiles may reveal underlying anxiety or ambivalence in certain contexts [50]. Interviewer lip corner-pullers in this context likely reflect a compensatory attempt to manage distress.

Our study contributes to growing empirical support for what has long been proposed by theoreticians: the clinician’s affective experience contains critical information about the patient [51-53]. Further, findings documented here call into question our tendency to focus exclusively on the verbal content in suicide assessments, and our tendency to focus exclusively on the patient. There is a great deal to learn from attending to the nonverbal experience of the clinical interviewer.

Importantly, this study sheds light on the use of machine learning–based tools for nonverbal behavior characterization. It is one of the first to examine the performance of an open-source, freely available software that uses deep learning to characterize facial and head behavior (ie, PyAFAR) on an entirely new dataset. Overall, performance of PyAFAR on our dataset can be considered acceptable for most AUs tested. Agreement between automated and manual coding about the absence of an AU was stronger than agreement about presence, and positive agreement rates were particularly low for the brow-lowerer, lip-depressor, and chin-raiser AUs. This is likely due to relatively low occurrence rates of these AUs in our reliability dataset. Additional intersystem reliability studies with longer video samples and therefore higher occurrence rates are needed to further assess agreement between automated coding and human coding. Still, there are many advantages of automated coding; human coding that took more than two years from training to completion could be produced in days by PyAFAR, with additional, rich information captured. Although PyAFAR does not produce results in real-time, its relative efficiency means that nonverbal behaviors can much more readily be incorporated into psychological research, addressing the current lack thereof that Hall et al [54] refer to as “a study of words without the music.” Our study demonstrates that automated coding has the power to efficiently detect clinically meaningful information exhibited via multiple nonverbal channels.

### Limitations and Future Research Directions

There are several limitations of this study. First, our sample, though sizable enough to detect effects, is small. Results of the prospective analyses on the suicidal subsample in particular should be interpreted with caution. Additionally, although our suicidal participants all reported past-year suicidal behavior, the degree of suicidality during the interview itself varied, and the number of participants with suicidal behavior at follow-up was small. Future research should seek to replicate this work with a larger sample and with more clinically acute participants, which would also enable a more fine-grained evaluation of possible sociodemographic differences in nonverbal behaviors (eg, by race and ethnicity, gender, education level). Relatedly, generalizability of study findings may be limited by the demographic makeup of our sample, which, while diverse with regard to race and ethnicity and sexual orientation, was predominantly female. There are also important limitations to our study design. All interviews were conducted via videoconference, and interviewers may differ in their ability to make emotional contact with a suicidal participant in this medium. Finally, this study is limited by its focus on each member of the dyad in isolation. Future research would benefit from conducting within-dyad analyses as well as between-group analyses to better understand the interactive processes that occur between interviewers and participants [24].

### Conclusions

This study documents the importance of attending to much more than just what patients say in considering suicide risk. Attending to nonverbal behavior holds the potential of improving suicide risk detection. Moreover, attending to the interviewer*,* as well as the patient, will also improve the study of suicide risk. Finally, the time-intensive nature of quantifying nonverbal behaviors through human coding is side-stepped by the approach to automated affective computing used here, which affords the opportunity to quantify nonverbal information efficiently and systematically, highlighting the potential of transdisciplinary, multimodal approaches in suicide research.

## Supplementary material

10.2196/85589 Multimedia Appendix 1 .Description of deviations from the preregistration.

## Abbreviations

AU: action unit
AUC: area under the curve
C-SSRS: Columbia-Suicide Severity Rating Scale
EAR: eye aspect ratio
FACS: facial action coding system
HIPAA: Health Insurance Portability and Accountability Act
LOOCV: leave-one-out cross-validation
MAR: mouth aspect ratio
PyAFAR: Python-Based Automated Facial Affect Recognition
QIDS: Quick Inventory of Depressive Symptomatology
ROC AUC: receiver operating characteristic area under the curve
SIQ: Suicidal Ideation Questionnaire
SVM: support vector machine
