# Supplementary material for: Using Automated Coding of Nonverbal Behavior During a Suicide Assessment to Inform Risk Detection: Mixed Cross-Sectional and Exploratory Prospective Study
Source: JMIR Form Res. 2026 Sep 16;10:e85589. doi: 10.2196/85589 (PMC13581149; doi:10.2196/85589)
Supplement: Supplementary file 1 [file formative-v10-e85589-s001.docx]

Our hypotheses and analysis plan were pre-registered on the Open Science Framework. We deviate from our pre-registration as follows:

(1) Our sample size of 70 was determined *a priori* to be able to detect medium effects with standard hypothesis testing. With capturing young adulthood in mind, we intended to focus on ages 18-24. However, in service of accelerating recruitment, we began with a broader age range of 18-26. The first 10% of participants (*n* = 7) run were recruited with this criteria, and one participant is therefore 26. Once we observed successful recruitment of younger adults, we reverted to 18-24 yrs. All other inclusion criteria remained the same throughout the study.

(2) We do not fine-tune the pre-trained model for Action Units (AUs) with s-scores <0.65 using our manually coded data due to functionality within PyAFAR. Instead, we raised the acceptable s-score threshold to 0.70, included additional measures to assess inter-systems reliability (AUC, F1, and negative agreement), and excluded AUs that did not reach this threshold from subsequent analyses (see *Inter-Systems Reliability*).

(3) We use the following summary statistics to characterize continuous time series data: mean, standard deviation, permutation entropy, change in quantile of mean, and complexity, due to recent research finding specific features to be especially predictive of depression severity and only extract these features on the subset of AUs for which PyAFAR provides intensity ratings.

(4) In addition to logistic regression, we assess performance of SVMs in predicting group membership given that SVM is better suited to small samples and examine and report results of both joint and separate participant/interviewer models (we do not include age and sex as predictors in these analyses as they were not related to suicide group status). Because we present performance metrics rather than significance testing at the variable level for our models, we do not include a correction for multiple comparisons.

(5) We include post-hoc exploratory analyses testing whether nonverbal behaviors were associated with suicidal thoughts and behaviors three months later in a subset of participants that completed a follow-up assessment (see *Post Hoc Exploratory Analyses*).
